# Effects of and Lessons Learned from an Internet-Based Physical Activity Support Program (with and without Physiotherapist Telephone Counselling) on Physical Activity Levels of Breast and Prostate Cancer Survivors: The PABLO Randomized Controlled Trial

**DOI:** 10.3390/cancers13153665

**Published:** 2021-07-21

**Authors:** H. J. van de Wiel, M. M. Stuiver, A. M. May, S. van Grinsven, N. K. Aaronson, H. S. A. Oldenburg, H. G. van der Poel, S. N. Koole, V. P. Retèl, W. H. van Harten, W. G. Groen

**Affiliations:** 1 Division of Psychosocial Research and Epidemiology, The Netherlands Cancer Institute, Plesmanlaan 121, 1066 CX Amsterdam, The Netherlands; h.vd.wiel@nki.nl (H.J.v.d.W.); m.stuiver@nki.nl (M.M.S.); n.aaronson@nki.nl (N.K.A.); s.koole@nki.nl (S.N.K.); v.retel@nki.nl (V.P.R.); w.groen@nki.nl (W.G.G.); 2 Center for Quality of Life, Netherlands Cancer Institute, Plesmanlaan 121, 1066 CX Amsterdam, The Netherlands; 3 Center of Expertise Urban Vitality, Faculty of Health, Amsterdam University of Applied Sciences, Tafelbergweg 51, 1105 BD Amsterdam, The Netherlands; 4 Julius Center for Health Sciences and Primary Care, UMC Utrecht, Utrecht University, P.O. Box 85500, 3508 GA Utrecht, The Netherlands; a.m.may@umcutrecht.nl; 5 Rijnstate Hospital, Wagnerlaan 55, 6815 AD Arnhem, The Netherlands; svangrinsven@rijnstate.nl; 6 Division of Surgical Oncology, The Netherlands Cancer Institute, Plesmanlaan 121, 1066 CX Amsterdam, The Netherlands; h.oldenburg@nki.nl; 7 Department of Urology, The Netherlands Cancer Institute-Antoni van Leeuwenhoek Hospital, Plesmanlaan 121, 1066 CX Amsterdam, The Netherlands; h.vd.poel@nki.nl; 8 Department of Health Technology and Services research, University of Twente, Drienerlolaan 5, 7522 NB Enschede, The Netherlands

**Keywords:** physical activity, Internet-based intervention, breast cancer survivors, prostate cancer survivors, RCT

## Abstract

**Simple Summary:**

Many cancer survivors have difficulties in attaining and maintaining physical activity (PA) after treatment. Therefore, we developed an Internet-based PA support program (IPAS), embedded in a patient portal. The aim of this study is to evaluate the effectiveness and costs of IPAS alone (online only) or IPAS combined with physiotherapist telephone counselling (blended care), compared to a control group. Our RCT included 137 breast and prostate cancer survivors. IPAS in its current form did not prove to be effective in increasing moderate to vigorous PA levels or secondary outcomes, compared to a control group, either as a standalone intervention or offered as blended care. We observed low to moderate satisfaction scores, with better scores for blended care. Recruitment and adherence to the study were challenging. Lessons learned led to suggestions for future trials, such as improved accessibility of the support program, increased frequency of support and use of activity trackers.

**Abstract:**

Background: We developed an Internet-based physical activity (PA) support program (IPAS), which is embedded in a patient portal. We evaluated the effectiveness and costs of IPAS alone (online only) or IPAS combined with physiotherapist telephone counselling (blended care), compared to a control group. Methods: Breast or prostate cancer survivors, 3–36 months after completing primary treatment, were randomized to 6-months access to online only, blended care, or a control group. At baseline and 6-month post-baseline, minutes of moderate-to-vigorous PA (MVPA) were measured by accelerometers. Secondary outcomes were self-reported PA, fatigue, mood, health-related quality of life, attitude toward PA, and costs. (Generalized) linear models were used to compare the outcomes between groups. Results: We recruited 137 survivors (participation rate 11%). We did not observe any significant between-group differences in MVPA or secondary outcomes. Adherence was rather low and satisfaction scores were low to moderate, with better scores for blended care. Costs for both interventions were low. Conclusions: Recruitment to the study was challenging and the interventions were less efficacious than anticipated, which led to lessons learned for future trials. Suggestions for future research are as follows: improved accessibility of the support program, increased frequency of support, and use of activity trackers.

## 1. Introduction

Higher levels of physical activity (PA) after cancer treatment are associated with lower levels of fatigue, better health-related quality of life (HRQOL) and mood, and better overall survival [1,2]. For example, in breast cancer, the leading cause of cancer in women, patients with high levels of post diagnosis PA showed a 29% and 39% reduction in risk of breast cancer-specific mortality and all-cause mortality, respectively, compared to those with low levels of PA [3]. In prostate cancer, the leading cause of cancer in men, high levels of physical activity were associated with a 38% reduction in risk for prostate cancer-specific mortality compared to the low levels of PA [4]. For this reason, supporting physical activity is becoming an increasingly important aspect of cancer care after medical treatment. Over the past years, systematic reviews have shown promising results of interventions aimed at improving physical activity levels [5,6], including e-health-based solutions in breast cancer [7], and prostate cancers patients and survivors [8]. Generally, supervised interventions have proven to be superior to unsupervised interventions [9]. However, in the light of the increasing number of cancer survivors, offering supervised interventions to all survivors puts a heavy burden on health care systems and society at large in terms of financial and human resources. In addition, the recent outbreak of COVID-19, which severely limited people’s opportunity to engage in supervised exercise, underscores the need to further develop and evaluate interventions that do not rely on in-person supervision. A meta-analysis on distance-based interventions, including a limited number of e-Health interventions, found that, overall, offering interventions remotely results in a small increase in minutes of MVPA (effect size = 0.2) [10]. Interventions in which eHealth and in-person or personal supervision are combined (so-called blended care) are appealing and are hypothesized to be more effective. They have therefore frequently been put forward as a research priority in multiple research fields [11,12,13]. 

To enable easy access for health care professionals, and to increase the perceived importance and clinical relevance of being physically active as a natural part of cancer care, we previously developed an Internet-based PA Support program (IPAS) as part of the implementation of a patient portal [14]. To the best of our knowledge, this was the first PA intervention that was built directly into a hospital patient portal. IPAS was developed in a stepwise manner, based on the Transtheoretical model (TTM) [15] and aspects of the Theory of Planned Behavior [16] and Social Cognitive theory [17]. Using focus group information and structured usability and feasibility testing in breast cancer survivors [18], the intervention was updated and extended to prostate cancer survivors.

The aim of the current study was to evaluate the effectiveness of the IPAS on PA levels of breast and prostate cancer survivors, and to explore the added value of blended care through monthly telephone support by a physiotherapist, compared to online only support and a control group. We hypothesized that additional physiotherapy counselling might increase adherence and effectiveness of the web-based intervention, based on reported benefits of low-level remote supervision by telephone in other RCT’s [19,20]. Although we focused primarily on the effectiveness of the interventions, we also investigated adherence to and satisfaction with IPAS both interventions. Based on our findings and on the lessons learned in conducting the trial, we also propose recommendations for future eHealth or blended care PA trials in the cancer survivorship setting. 

## 2. Materials and Methods

A detailed description of the trial protocol and interventions has been published elsewhere [21]. The differences between the protocol paper and the current paper comprise the following: (1) We did not reach the intended inclusion numbers (*n* = 246, for more details of the sample size calculation we refer to the design paper [21]), due to time and budget constraints as well as lower than anticipated numbers of eligible patients; (2) in an effort to increase the number of participants, the inclusion criterion of being between 3 and 12 months post-primary treatment was expanded to 3–36 months; (3) due to planning and budget constraints, IPAS was not built into the patient portal of the University Medical Center Utrecht the (UMCU); instead, patients of the UMCU were invited to use IPAS of the Netherlands Cancer Institute (NKI) and therefore clustered with NKI in the minimization procedure; and (4) we performed a cost-analysis instead of the cost-effectiveness because of the null findings. 

### 2.1. Research Design and Study Population

The PABLO-trial was a multicenter randomized controlled trial of a mostly distance-based intervention to promote physical activity levels. Breast and prostate cancer survivors were randomized into three groups; (1) online only: IPAS (2) blended: IPAS + additional telephone support from a physical therapist, or (3) control group. Patients were allocated to one of the three study arms using a minimization procedure that ensured balance between the groups in terms of tumor type (breast/prostate), hospital (NKI/Rijnstate), age (>50, 50–60, >60), and current endocrine treatment (yes/no). Participants were randomized by the researcher, using a computer-generated random assignment procedure with a 1:1:1 distribution (ALEA, [22]), assuring blinded treatment allocation. Neither participants nor researchers were blinded to the randomization results. 

Breast and prostate cancer survivors who had completed primary curative treatment 3–36 months earlier, but who could still be under adjuvant endocrine treatment or Trastuzumab, were invited to participate in the PABLO-trial. Potentially eligible survivors were excluded in case of lack of basic proficiency in Dutch, serious cognitive or psychiatric problems that would preclude following the intervention or completing the study questionnaires, and lack of Internet access. Patients without a digital ID (DigID)—the Dutch digital authentication system based on one’s social security number (mostly used for governmental services)—were also excluded, as this was required to log on to the IPAS. Moreover, patients participating in concurrent studies or rehabilitation programs containing psychosocial and/or exercise interventions were excluded, as well as patients who were unable to perform unsupervised exercise at the recommended levels or who could not safely perform such exercise according to the pre-exercise screening recommendations of the American College of Sports Medicine [23]. Patients with cardiovascular, metabolic, or renal disease could only participate after approval by their treating physician. Lastly, to start with sufficient contrast, we excluded patients who already reported to be engaging in >200 min MVPA/week of moderate to vigorous PA for more than 6 months, as assessed via a short interview.

### 2.2. Procedure

**Figure 1:**
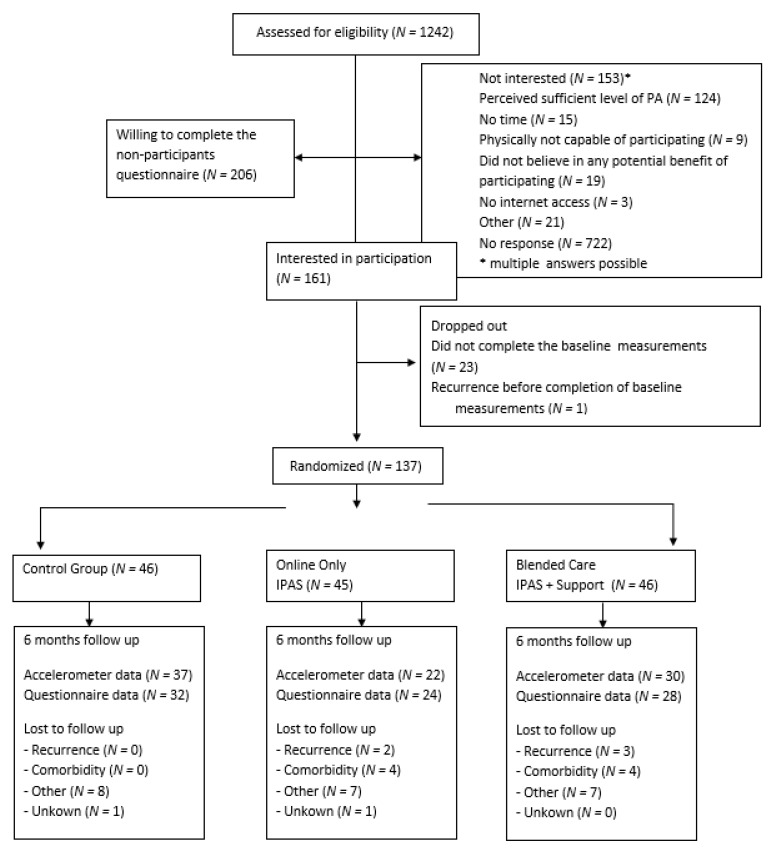
CONSORT diagram PABLO trial.

The recruitment strategy involved either in-person or mail-based introduction to the study: (1) The treating medical specialist or specialized nurse practitioners informed the patient about the study during a follow-up appointment in the hospital. The clinician checked eligibility with the aid of a screening-list of inclusion and exclusion criteria. Interested patients received an information package. After two weeks, the researcher contacted the patient by phone to confirm whether or not (s)he was willing to participate; or (2) eligible patients were identified from medical records by the treating physician and were sent study information by mail. If approved via a response card, the researcher contacted the patient by phone to confirm participation or non-participation. Figure 1 shows the CONSORT-EHEALTH diagram [24] of the trial.

All participants in the current study provided written informed consent and completed the questionnaire online. Ethical approval was obtained from the institutional review board of the Netherlands Cancer Institute, Amsterdam (NL62269.031.17).

### 2.3. Trial Arms

The control group received usual care with an additional printed leaflet on guidelines and possible benefits of PA after cancer treatment. The leaflet presented the Dutch physical activity guidelines and provided information on how participants could monitor intensity of their PA using the Borg rating of perceived exertion, a “talk-test” (when it becomes more difficult to talk in whole sentences), and heart rate self-monitoring.

Online only patients who were randomized to the online only intervention group received access to IPAS. IPAS is structured according to the Transtheoretical model (TTM) [15] and uses aspects from the Theory of Planned Behavior [16] and Social Cognitive theory [17]. The TTM postulates that subjects can be classified into one of five stages of behavioral change related to the desired behavior, in this case, meeting physical activity guidelines [15]. Accordingly, the five stages identified by IPAS were (1) precontemplation (not sufficiently active and not intending to change), (2) contemplation (not sufficiently active but willing to change within next 6 months), (3) preparation (not sufficiently active but planning to change within 1 month), action (sufficiently active but for less than 6 months) or maintenance (sufficiently active for longer than 6 months). Patients could move through these stages during the intervention. In every stage, patients received information, images, and interactive assignments and videos that matched their current stage. During the six months of the intervention, patients received emails prompting them to complete an online questionnaire querying current PA behavior and stage of change. These emails were sent one month after completing the previous questionnaire. If patients forgot to complete the questionnaire, a reminder was sent after one week. After patients completed their questionnaire, they were directed to a content page in IPAS that provided feedback by benchmarking patients’ current PA levels against the cancer survivorship guidelines (i.e., ≥150 min moderate to vigorous physical activity per week and two days a week of muscle strengthening exercises), using a graph and a table [25]. New material was provided monthly, tailored to the patients’ stage of change.

Blended patients who were randomized to the blended care group also received access to the IPAS intervention, but additionally received a face-to-face intake at baseline, and monthly phone calls from a physiotherapist. During the 45-min intake, the physiotherapist briefly introduced the study and the IPAS intervention, and discussed current PA level, motivation, and barriers to PA, as well as possible strategies to deal with identified barriers. Subsequently, the patient was asked to exercise on a stationary bike or treadmill, to experience the desired moderate intensity. The intake was concluded by setting a clear goal with the patient for the intended behavior change, and by establishing the most convenient time at which the patient could be reached by telephone. Telephone calls were scheduled monthly. During the monthly call, the physiotherapist first confirmed the stage of change by discussing current PA levels. Next, the physiotherapist provided feedback on the stage of change and reminded the patient of the goals set during the previous (intake- or telephone-) consultation. The physiotherapist then helped the patient reflect on their experiences and accompanying thoughts, and normalized, reinforced, or explained physical activity behavior, whenever relevant. The telephone consultation concluded with setting a new behavioral goal (related to physical activity) for the next month. Physiotherapists took notes on each discussion in standardized forms. All physiotherapists received training and detailed guidelines for the intake and telephone calls.

### 2.4. Measurements

All outcomes were assessed at baseline, post-intervention (6 months) and at 12 months follow-up. Here we report on the post-intervention effects at 6 months.

### 2.5. Primary Outcome

The primary outcome was change in weekly time spent in MVPA, as measured with an accelerometer, the Actigraph GT3X+ activity monitor (Actigraph, Pensacola, FL, USA) worn for seven consecutive days, at baseline and after 6 months. The Actigraph is a small tri-axial accelerometer that can measure accelerations from 0.05 to 2.00 G [26]. An instruction leaflet was provided to explain how the device should be worn on the right hip.

Accelerometer data were processed using the ActiLife software program (ActiLife, Pensacola, FL, USA). Valid days were defined as days with at least 600 min of wear time; non-wear time was defined as 60 min of consecutive zero counts [26]. We included participants in the analyses who had at least four valid weekdays and one valid weekend day [27]. Weekly time spent in MVPA was then calculated by standard cut-points [27,28,29].

### 2.6. Secondary Outcomes

Secondary outcomes were changes in self-reported level of PA as assessed by the International Physical Activity Questionnaire [30], fatigue as assessed with the Multidimensional Fatigue Inventory [31], mood as measured with the Profile of Mood States [32], HRQOL as assessed with the 36-Item Short Form Health Survey and the EuroQol EQ-5D-5L [33,34], and attitude and behavior toward PA using specific items as used in previous studies [20,35,36,37,38,39]. In addition, we performed a cost-analysis [40,41].

A detailed description of the questionnaire characteristics and methods of the cost-analyses is available in the Supplementary Materials (Supplementary Material Sa) [21].

### 2.7. Statistical Analyses

#### 2.7.1. Sociodemographic and Clinical Data

Descriptive statistics were generated for baseline data to characterize and compare the intervention and control groups in terms of clinical and sociodemographic characteristics. Clinical data of interest, including tumor type and staging, type of treatment(s) and time between diagnosis and the end of primary treatment, were obtained from the medical records. Sociodemographic information about age, sex, educational level, living and work situation, as well as lifestyle data such as smoking behavior, alcohol consumption, and PA behavior prior to the cancer diagnosis were assessed with the T0 questionnaire. The questionnaire also included study-specific questions about patients’ use of the Internet and their level of computer skills. Data cleaning and analyses were performed in the R statistical package (3.3.1, [42]) using the Rstudio interface (Version 1.1453, 2009–2018, Rstudio Inc., Boston, MA, USA).

#### 2.7.2. Primary Outcome

We evaluated differences over time in minute of MVPA between both interventions and the control group with generalized linear regression models adjusted for MVPA baseline values and minimization factors.

#### 2.7.3. Secondary Outcomes

We evaluated between-group differences in self-reported levels of PA, fatigue, mood, and HRQOL using linear regression models, adjusted for baseline values of the outcome variables and minimization factors. Scores for the IPAQ, MFI, POMS, and SF-36 were calculated according to existing algorithms. We report adjusted mean differences between the separate and combined intervention groups and the control group. A *p*-value of 0.05 was considered to indicate statistical significance. All analyses were conducted on an intention-to-treat basis. We did not correct *p*-values for multiple testing.

#### 2.7.4. Adherence

The majority of previous studies used adherence cut-offs of 66% for eHealth interventions [43,44]. Considering the relatively low total number of log-ins and/or support calls required, we decided on a higher threshold and defined adherence with the intervention as a minimum of 5 log-ins (80%) for the IPAS-only group, and 80% of the intended log-in frequency or completing 80% of the five scheduled calls with the physical therapist for the blended care group.

## 3. Results

Of the 1242 invited patients, 137 participated in the PABLO-trial (participation rate: 11%), as displayed in the CONSORT diagram of Figure 1. Baseline characteristics are presented in Table 1.

### 3.1. Effect on Primary Outcome

No statistically significant between-group differences were observed in minutes of MVPA per week between the online only group and the control group (β −15.42 (95% CI = −51.5:15.6), *p* = 0.39), nor between the blended care group compared to the control group (β 5.70 (95% CI = −30.5:37.6), *p* = 0.75) (Table 2). Analyses per tumor type did not show significant differences in MVPA minutes per week. Additionally, when both intervention groups were combined, we did not observe a significant difference in MVPA minutes per week when compared to the control group (β −1.99 (95% CI = −38.5:28.0, *p* = 0.91).

### 3.2. Effect on Secondary Outcomes

Detailed results of the secondary outcomes are presented in the supplementary data section. In short, we did not find statistically significant differences between groups for the secondary outcomes self-reported physical activity, fatigue, mood, self-efficacy, or behavioral and attitudinal variables toward PA, with the exception that the blended care group reported significantly higher scores on the MFI subscale “reduced activity” (β 2.33 (95% CI = 0.1:3.1), *p* = < 0.01) and for the SF36 subscale “bodily pain” (β −12.09 (95% CI = −20.3:−3.9), *p* = < 0.01) compared to the control group. Analyses per tumor type did not show noteworthy differences between the groups. When both intervention groups were combined, we only observed a significant difference on the SF-36 subscale “mental health” in favor of the combined intervention group (β 4.4 (95% CI = 0.1:8.8), *p* = 0.046). No other significant differences were observed.

### 3.3. Actual Use and Adherence

Of the 22 participants of the online only group, seven never logged into the intervention. The remaining 15 logged in, on average, 1.6 times (median: 1; range 1 to 8).

Of the 25 participants of the blended care group, 10 never logged in, and 2 stopped the study before the intake with the physiotherapist. The remaining 13 logged into the online portal a mean of 2.5 times (median: 2; range 1 to 9). The median number of phone calls with the physical therapist was 3 (range 0 to 6).

In total, 40 (15 online only, 25 blended) (47.1%) participants were classified as “users” of the interventions, as they logged in two or more times. Two participants of the online only group reached 80% adherence (logged in four times or more), and seven blended-care participants reached an 80% adherence to both elements. Fourteen participants (56%) of the blended group reached 80% adherence with regard to physiotherapist support, defined as four successful calls; 11 participants (44%) were contacted by phone fewer than three times.

### 3.4. Per Protocol Analysis of the Primary Outcome

A post-hoc *per protocol* analysis, including only the participants of the intervention groups who were at least 80% adherent, did not change the conclusion of the intention-to-treat analysis. In this analysis, we found no statistically significant differences in minutes of MVPA per week between the only group and the control group (β 50.3 (95% CI = −141.0:241.5), *p* = 0.60), nor between the blended care group compared to the control group (β 9.4 (95% CI = −79.0:97.9), *p* = 0.83).

### 3.5. Evaluation of the Intervention

The evaluation questions show low to moderate scores on usefulness, usability, and satisfaction with the intervention. In general, the blended care group seems to be more positive about all components of the intervention, even though they experienced more technical issues. Lastly, the majority of the blended care group that logged in, would recommend it to other survivors (Table 3).

### 3.6. Cost-Analysis

Clinical utility scores calculated from the EQ-5D-5L questionnaire for patients with prostate cancer and breast cancer, are listed Supplementary Material Table S3a,b, respectively. Assuming that the intervention is offered to 1000 patients annually, estimated additional costs for implementation of the IPAS online intervention are €14.16 per patient per year. When combined with the physiotherapists’ intervention (blended care), estimated additional costs are €140.19 per patient per year (Supplementary Material Table S2 and Figure S1).

## 4. Discussion

In this RCT carried out among breast- and prostate cancer survivors, we did not observe significant differences in PA levels between those who received an Internet-based PA program, offered online or via blended care, compared to the control group. Additionally, no noteworthy significant differences were observed for the secondary outcomes such as fatigue, mood, or HRQOL. Although promising results have been reported (7, 8), our findings are not unique, as there are many distance-based intervention studies which have failed to substantially increase PA levels [10,45]. Previous studies critically discussed the importance of a well-considered design, technology, and delivery methods to enable adherence and effectiveness of web-based interventions [46,47,48,49]. We carefully considered all of these issues, and described the stepwise, evidence-, and pilot-based development of IPAS in the introduction. Here, we take a closer look at these choices in terms of innovative technology and delivery modes, and how they might have influenced the results of the current trial. We will discuss these consequences and other issues that we faced during the trial in terms of intervention design, recruitment, and adherence. Based on the lessons learned, we propose recommendations to further advance the field of e-health research.

### 4.1. Intervention Design

The embedding of IPAS into the online patient portal was an important choice in the design of the intervention, as it increases the likelihood of integrating such a program into the hospital’s patient-management pathway (Supplementary Material Table S1). Although this decision was well thought through, and based on demonstrated feasibility in a pilot study [18,50], we experienced a number of setbacks related to this choice. First, although the lay-out and tailoring of the webpages were significantly improved since the pilot study, the portal software still had limited graphical possibilities, e.g., graphs were quite rudimentary in their visual display. Second, due to privacy issues, the log-in was restricted to a single person (i.e., the participating survivor), which limited real-time testing and monitoring options for the research team members during the intervention. This dependency on feedback of participants made it difficult to detect early on possible technical issues with use of the program by individual participants throughout the course of the trial. In addition, the access via a personal identification log-in (DigID) may have represented a barrier to log-in because of the required multi-factor authentication. Third, although the patient portal was accessible on mobile phones, the layout did not scale and hence it was only optimal when accessed via a computer or laptop; this did not accommodate patients’ increasing preference for accessing Internet content via their smartphone.

### 4.2. Recruitment

Recruitment strategy and societal changes should be taken into account when interpreting the results. We observed a lower inclusion rate (11%) when compared to previous (semi-)supervised exercise oncology trials, which reported rates of about 40% [37,51,52,53]. However, it is difficult to compare those rates with ours, since we invited patients via their treating physician based only on their clinical information in the medical records, and we were not able to screen survivors on PA-levels before sending the invitation. This in turn led to approaching many survivors who, in fact, were not eligible for participation because they reported sufficient PA levels. Moreover, the low recruitment rates could be explained by societal changes over the past years. For instance, due to guidelines for physical activity [54], there is increasing emphasis on and awareness of the need for an active lifestyle, which has led to less eligible patients. Although we did not reach our intended number of participants and were thus unpowered to detect our hypothesized outcomes, the results do not show any trends of beneficial effects. It is unlikely that a fully powered trial would have led to other conclusions.

As soon as we saw that the recruitment numbers were lower than anticipated, next to expanding the inclusion criteria, we considered expanding the trial to include more hospitals and other cancer types. Due to the choice to embed IPAS into the patient portal, we faced high costs for incorporation of IPAS in the hospital’s portal, in addition to ICT planning difficulties caused by the need to employ specifically trained personnel to build and update the intervention into the patient portal of other hospitals. Consequently, extension to another hospital was not possible in view of their IT project calendar and deemed too costly, and could therefore not take place. Furthermore, one of the hospitals of the multicenter trial (Rijnstate) started the trial, but switched to an updated EMR with a next version of a patient portal system after a short period of accrual. As IPAS was not compatible with the new portal, recruitment in that hospital had to stop approximately one year earlier than intended. We considered also making IPAS suitable for other (less prevalent) cancer types as a means of increasing accrual rates. However, since IPAS was content-specific for breast- and prostate cancer, the costs and time required to adjust the intervention to other cancer types did not outweigh the slightly increased recruitment rate expected in the remaining timeframe of the current study.

### 4.3. Adherence

In line with a systematic review on adherence to web-based interventions [55], we observed low adherence rates. The accelerometer-based baseline level of MVPA of our sample was already high, despite our initial screening (baseline mean 281 MVPA min/week), and slightly higher than in a previous post-treatment study in breast (mean 238 MVPA min/week) [53] and prostate cancer survivors (mean 266 MVPA min/week) [56]. This could be explained by selective participation of highly educated patients and the increased awareness of the importance of PA nowadays in both the general population as well as in health care settings, in combination with possible underreporting of PA-levels at the initial screening for eligibility. The intervention was developed with the intention to target relatively inactive survivors, and included a variety of strategies to increase self-efficacy and improve physical activity behavior. Although we screened the participants before study-entry, 50% of them reported to be in the maintenance phase. This means half of our participants considered themselves to be sufficiently active and able to maintain this behavior, in addition to reporting relatively high levels of self-efficacy. As a result, the participants to the study may not have been very receptive to the strategies applied by IPAS. Additionally, the low adherence rates can be explained, in part, by issues that arose as a result of the integration of IPAS into the patient portal. To increase adherence to the program, for the delivery of e-mails, we included automated invitation e-mails to log into IPAS, and reminders that had to be sent a week after the monthly invitation. However, for a period of approximately four months, these e-mails were not sent consistently due to technical issues. Incidental, individual technical problems with e-mails and videos occurred as well. Due to the limited test-abilities of the portal as discussed above, we discovered these problem relatively late. This could have negatively influenced adherence during that time and might have been prevented if we had tested the IPAS in a mock-up setting. Given the low adherence [57], intervention fidelity problems that led to suboptimal effects should be considered due to technical issues [58]. The low adherence rates may have reduced the contrast between the intervention groups and the control group, even more so because the control group actively received a leaflet with an extended description of current PA guidelines.

### 4.4. Physical Activity Support by Blended Care

As mentioned in the introduction, although intuitively appealing, little is known about the effectiveness of blended care relative to full e-Health interventions, or about the intensity of added in-person support that is needed to achieve optimal results [47]. The monthly frequency of our blended interaction was reported as “low” by many participants. The six involved physiotherapists experienced four situations in their intake or consultations: some patients expected more specific advice on the intervention, e.g., a detailed outlined exercise program. Some had a difficult time using IPAS, due to technical issues and/or lack of self-management skills. Others experienced the telephone calls more as “monitoring/judging,” rather than as a supportive moment, despite the fact that physiotherapists were instructed not to be judgmental in their communication. This phenomenon was also observed in a previous study [43]. Nevertheless, there were also patients who experienced the telephone consults as helpful. Overall, the majority of participants would recommend the program to others, despite the shortcomings. However, the bottom line is that the blended care support as employed in our trial, with monthly contact, did not increase the efficacy of the online intervention. Further research of online only compared to different intensities of blended care is needed to gain more insight into an optimal delivery method of PA interventions.

In addition to the limitations of the study as described above, the COVID19 lockdown during our measurement phase should be noted. Although eHealth interventions during such a lockdown could be considered unique opportunities, IPAS was not specifically designed to support PA under circumstances in which the population as a whole self-reported a decrease in PA levels during the pandemic [59,60,61]. For example, many of the suggested strategies to increase physical activity relied on opportunities to do physical activity outside, or together with peers. Such opportunities were limited during the lockdown.

Our study also has several strengths worth noting. The first obvious strength is the randomized controlled design which is considered as the gold-standard in intervention research [58]. In addition, the study included a head-to-head comparison of different delivery methods for remote support of PA. Furthermore, we measured PA objectively with an accelerometer for seven consecutive days, which eliminated recall bias. Finally, we included a costs analysis, which shows that the intervention is relatively inexpensive (<15 euro per patient if offered to at least 1000 patients). At this low price, it could be easily offered to patients on a hospital-wide level.

### 4.5. Future Directions

Based on the lessons learned, we would make a number of recommendations for future trials and interventions in this field. Despite the theoretical advantages from a cognitive behavioral point of view, and in view of the commitment of clinicians and nurses, we would advise against the embedding of interventions into a secure patient portal as long as a number of design and implementation obstacles have not been adequately solved. This includes both ensuring the availability of sufficiently flexible and sophisticated software programs and having sufficient time to build, test, optimize, and integrate such programs into the ICT environment of all study sites before the start of the trial. If it is not possible, due to costs and/or operational barriers, to integrate such programs into existing patient portals, it may be more sensible to develop and test a stand-alone version that offers more flexibility and makes fewer demands hospital patient portal systems [8]. A stand-alone website or app, which can be more easily developed, could be implemented and maintained at relatively lower costs. More frequent updates, assignments and reminders, and a user interface that automatically scales to mobile device screens could increase usability and adherence [55]. However, unless the data are collected anonymously, authorization regulations should still be taken into account in stand-alone interventions. Finally, although a Cochrane review concluded that supervision is important in ensuring adherence to PA interventions [6], our findings suggest the need for a more intensive blended care component than used in the current trial. The use of relatively inexpensive PA-tracking technologies (e.g., apps and wearables) can potentially facilitate (self)monitoring and thus increase adherence to and efficacy of the PA interventions [48,62,63,64].

## 5. Conclusions

The IPAS intervention in its current form did not prove to be effective in increasing moderate to vigorous PA levels or secondary outcome variables, compared to a control group, either as a standalone intervention or offered as blended care. Limitations due to the embedding of the program into the patient portal and dependence on hospital IT choices led to poor recruitment and indirectly to low adherence rates. Future interventions should be more easily accessible and more frequent support should be considered for blended variants. Additionally, the interventions should also speak to the more PA-aware survivors. Finally, adding tracking wearables could potentially improve the program adherence and monitoring. Future research should focus on determining the nature and intensity of the blended care supervision needed to improve the efficacy and cost-effectiveness of Internet-based PA interventions.

## Figures and Tables

**Table 1:** Baseline characteristics.

Characteristics	Control Group *N =* 46	Online Only (IPAS) *N =* 45	Blended Care (IPAS + Support) *N =* 46
**Tumor type/sex***N* (%) Prostate cancer/Men Breast cancer/Women	24 (52) 22 (48)	22 (49) 23 (51)	24 (52) 22 (48)
**Center***N* (%) AVL Rijnstate UMCU ^a^	42 (91) 3 (7) 1 (2)	42 (93) 3 (7) 0 (0)	41 (89) 3 (7) 2 (4)
**Treatment***N* (%) ^b^			
Radiotherapy Prostate cancer Breast cancer	8 (35) 16 (73)	5 (23) 16 (70)	6 (25) 16 (73)
Chemotherapy (only in breast cancer) (Neo)adjuvant Adjuvant	14 (31) 4 (9)	6 (13) 3 (7)	5 (11) 3 (7)
Endocrine therapy Prostate cancer Breast cancer	2 (9) 13 (59)	1 (5) 10 (44)	2 (8) 9 (41)
Breast-conserving surgery ^c^	15 (68)	14 (61)	12 (55)
Mastectomy ^c^	7 (32)	7 (30)	10 (46)
Breast reconstruction ^c^	8 (36)	8 (35)	8 (36)
Prostatectomy ^d^	16 (70)	20 (91)	18 (77)
Brachytherapy ^d^	0 (0)	0 (0)	2 (8)
**Treatment duration** in months (median, IQR) Prostate Breast	3 (2.5–9) 6 (5–8.5)	2.5 (2–5) 4 (4–8)	3 (3–6.5) 7 (5.5–9.5)
**Age** in years (mean, SD)	59.2 (14.4)	59.3 (11.3)	59.8 (11.7)
**Living situation (%)**			
Single	7 (15)	6 (13)	10 (22)
Living together	36 (78)	38 (84)	34 (74)
With partner, not living together	2 (4)	1 (2)	2 (4)
Missing	1 (2)	-	-
**Education level***N* (%)			
Primary school	1 (2)	0 (0)	1 (2)
High School	12 (26)	14 (31)	19 (41)
College/University	31 (68)	31 (69)	25 (54)
Missing	2 (4)	-	1 (2)
**Working situation***N* (%) ^e^			
Paid Job	15 (33)	21 (47)	22 (48)
Retired	17 (37)	13 (29)	15 (33)
On disability	8 (17)	4 (9)	5 (11)
**Smoking behavior***N* (%)			
Never	24 (52)	18 (40)	20 (44)
Previous	18 (39)	25 (56)	21 (46)
Current	3 (7)	2 (4)	5 (11)
Missing	1 (2)	-	-
**Alcohol consumption***N* (%)			
No	12 (26)	13 (29)	10 (22)
Yes	33 (72)	32 (71)	36 (78)
Missing	1 (2)	-	-
**Computer use***N* (%)			
Sometimes	4 (9)	2 (4)	1 (2)
Often	41 (89)	43 (96)	45 (98)
Missing	1 (2)	-	-
**Computer skills** *N* (%)			
Poor	3 (7)	5 (11)	3 (7)
Moderate	15 (33)	8 (18)	14 (30)
Good	27 (58)	32 (71)	29 (63)
Missing	1 (2)	-	-
**Physical activity levels before diagnosis** *in days per week* (*median, IQR*)			
*Moderate ^f^*	5 (4–5)	5 (4–6)	6 (4–6)
*Vigorous ^g^*	2 (1- 2)	2 (2–3)	1 (1–3)

^a^ Recruited at University Medical Central Utrecht (UMCU), patients used the IPAS from AVL; ^b^ Combination of treatments possible per patient, total percentages reaches above 100%; ^c^ percentage of women, control *n* = 22, online only *N* = 22, blended *N* = 21; ^d^ percentage of men, control *n* = 23, online only *N* = 22, blended *N* = 24; ^e^ multi answer options, total percentages reaches above 100%; ^f^ question: How many days of the week have you been moderately physically active for at least 30 min last week? ^g^ Question: How many days of the week have you been vigorously physically active for at least 20 min last week?

**Table 2:** Primary outcome.

	T0 (*N* = 84)	T1 (*N* = 84)	Between Group Differences	
Measure	Mean (SD)	Mean (SD)	AMD ^†^ (95% CI)	*p*-Value
Primary Outcome				
**MVPA/week** Control*)*^‡^ *(N =* 34*)* Online Only *(N =* 21*)* Blended *(N =* 28*)*	289.5 (128.2) 309.4 (152.6) 245.6 (147.4)	294.8 (162.7) 291.9 (142.1) 240.3 (156.8)	REF −15.42 (−30.5:37.6) 5.70 (−30.5:37.6)	0.39 0.75

^†^ AMD: adjusted mean difference. ^‡^ REF: control is reference group.

**Table 3:** Evaluation IPAS.

	Online Only *N* = 22 * (Mean, SD)	Blended *N* = 25 * (Mean, SD)
Technical issues (*N,* % yes)	2 (8)	9 (36)
I experienced IPAS as useful ^a^	2.8 (1.2)	3.2 (1.1)
I experienced the role of physiotherapist as useful ^a^	NA	3.7 (0.7)
I experienced IPAS helpful in becoming more physically active ^a^	2.3 (1.1)	3.3 (1.0)
The physiotherapist helped me to become more physically active ^a^	NA	3.5 (1.0)
I am satisfied about IPAS ^a^	2.7 (0.9)	2.9 (0.9)
I am satisfied about the role of physiotherapist ^a^	NA	3.5 (0.8)
IPAS and the physiotherapist strengthen each other’s effect ^a^	NA	3.3 (0.8)
I would value IPAS on a scale from 1 till 10 ^b^	5.4 (2.5)	6.1 (1.5)
I would recommend IPAS to other cancer survivors (*N*, %) Yes Maybe No	3 (14) 15 (68) 4 (18)	13 (52) 10 (40) 2 (8)

* Online only: missing = 2 due to incomplete questionnaire; blended: missing = 3 due to incomplete questionnaire; ^a^ assessed on a 5-point Likert scale (fully disagree, disagree, not sure, agree, fully agree); ^b^ assessed on a 10-point scale.

## Data Availability

Data available on request due to restrictions e.g., privacy or ethical. The data presented in this study are available on request from the corresponding author. The data are not publicly available due to privacy and ethical restrictions.

## Supplementary Materials

The following are available online at https://www.mdpi.com/article/10.3390/cancers13153665/s1, Figure S1: Total costs for both interventions calculated per patient, dependent on patient volume, Table S1: Advantages and Disadvantages of the embedding into the patient portal, Table S2: Costs PABLO interventions, Table S3: Summary scores per tumor type, Table S4: Secondary outcomes.
