# Supplementary material for: Effects of and Lessons Learned from an Internet-Based Physical Activity Support Program (with and without Physiotherapist Telephone Counselling) on Physical Activity Levels of Breast and Prostate Cancer Survivors: The PABLO Randomized Controlled Trial
Source: Cancers (Basel). 2021 Jul 21;13(15):3665. doi: 10.3390/cancers13153665 (PMC8345041; doi:10.3390/cancers13153665)
Supplement: Supplementary file 1 [file cancers-13-03665-s001.zip › SupplementaryDataSection.pdf]

Supplementary Data section:

Table S4. Secondary outcomes.

	T0 (N=84)	T1 (N =84)	Between group differences	
**Measure**	Mean (SD)	Mean (SD)	AMD† (95% CI)	P- value
		*Secondary Outcomes**		
		**IPAQ A – intensity**		
**IPAQ A – walking**				
Control ‡	1607.7 (3405.1)	1378.8 (1519.8)	REF	
Online Only	1367.4 (1383.8)	1050.8 (1088.5)	-136.57	0.79
Blended	2889.5 (6582.1)	1583.4 (2648.7)	36.48	0.94
**IPAQ A – Moderate physical activity**				
Control ‡	2800.3 (3390.4)	2733.6 (3154.9)	REF	
Online Only	4222 (5904.7)	3123.7 (2980.8)	-7.20	0.99
Blended	3834.1 (3924.3)	4098.6 (4670.5)	1159.17	0.22
**IPAQ A – Vigorous physical activity**				
Control ‡	833.8 (1438.7)	1226.3 (2049.0)	REF	
Online Only	1528.3 (4108.6)	1596.7 (4249.4)	-104.26	0.82
Blended	1604.3 (3095.3)	881.1 (1287.9)	-837.36	0.06
		**IPAQ A – per domain**		
**IPAQ A – work**				
Control ‡	565.5 (1301.7)	1046.6 (2744.7)	REF	
Online Only	2439.3 (6049.3)	2146.8 (4798.1)	-170.88	0.78
Blended	2461.8 (5538.3)	1743.3 (4023.1)	-550.10	0.35
**IPAQ A – at home**				
Control ‡	1570.6 (2275.9)	1818.8 (2741.8)	REF	
Online Only	1802.7 (3004.6)	1051.7 (1355.2)	-0.00	0.13
Blended	2463.0 (4020.5)	2226.4 (3303.0)	0.04	0.52
**IPAQ A – leisure time**				
Control ‡	1522.3 (2071.6)	1256.5 (1544.2)	REF	
Online Only	1225.1 (1931.5)	1222.4 (1480.7)	-119.96	0.76
Blended	1345.3 (2305.9)	1140.1 (1187.0)	8.03	0.98
**IPAQ A – during transport**				
Control ‡	1474.9 (2227.7)	1216.8 (1346.3)	REF	
Online Only	1244.2 (1438.7)	990.2 (1174.6)	-171.73	0.61
Blended	2643.6 (6536.3)	1209.3 (1205.9)	-195.37	0.54
**MFI B: General fatigue**				
Control ‡	11.6 (4.2)	10.7 (3.7)	REF	

Online Only	10.1 (4.5)	9.1 (4.5)	-1.15	0.22
Blended	11.8 (4.5)	11.8 (4.2)	0.87	0.31
**MFI B: Physical fatigue**				
Control ‡	12.0 (4.5)	10.1 (4.7)	REF	
Online Only	10.3 (4.4)	8.9 (4.8)	-0.21	0.82
Blended	11.1 (4.3)	10.8 (4.0)	1.22	0.15
**MFI B: Reduced activity**				
Control ‡	11.8 (3.4)	10.0 (3.6)	REF	
Online Only	10.3 (4.4)	10.1 (4.2)	0.63	0.49
Blended	10.3 (4.3)	10.3 (4.3)	2.33	< 0.01
**MFI B: Mental fatigue**				
Control ‡	10.2 (4.1)	8.8 (3.7)	REF	
Online Only	9.5 (3.9)	7.8 (4.3)	-0.47	0.62
Blended	9.5 (3.7)	9.5 (4.6)	0.72	0.41
**MFI B: Reduced motivation**				
Control ‡	10.2 (2.9)	9.6 (3.7)	REF	
Online Only	9.1 (3.4)	8.2 (3.6)	-1.22	0.14
Blended	8.5 (3.4)	9.4 (3.1)	0.73	0.34
**POMS C - Fatigue**				
Control ‡	1.7 (0.7)	1.8 (0.9)	REF	
Online Only	1.5 (0.8)	1.6 (0.6)	-0.19	0.43
Blended	1.9 (1.0)	2.0 (1.0)	-0.00	0.99
**POMS C - Tension**				
Control ‡	1.6 (0.8)	1.7 (0.9)	REF	
Online Only	1.3 (0.6)	1.4 (0.6)	-0.16	0.46
Blended	1.7 (0.9)	1.7 (0.9)	-0.05	0.82
**POMS C - Depression**				
Control ‡	1.4 (0.6)	1.6 (0.9)	REF	0.12
Online Only	1.2 (0.6)	1.2 (0.4)	-0.33	0.27
Blended	1.6 (0.9)	1.5 (0.8)	-0.22	
**POMS C - Anger**				
Control ‡	1.4 (0.5)	1.5 (0.9)	REF	
Online Only	1.2 (0.5)	1.2 (0.3)	-0.29	0.15
Blended	1.6 (0.9)	1.6 (0.9)	-0.09	0.64
**POMS C - Vitality**				
Control ‡	3.3 (0.9)	3.2 (0.8)	REF	
Online Only	3.8 (0.9)	3.7 (0.9)	0.44	0.08
Blended	3.4 (0.9)	3.3 (0.8)	0.15	0.52
**POMS C - Total**				
Control ‡	2.8 (2.7)	3.5 (3.8)	REF	
Online Only	1.5 (2.4)	1.7 (2.4)	-1.54	0.13
Blended	3.6 (3.9)	3.5 (3.7)	-0.22	0.81
**SF-36 D- Physical functioning**				

Control ‡	80.6 (19.6)	85.5 (16.4)	REF	
Online Only	88.5 (13.1)	91.7 (9.0)	1.07	0.67
Blended	84.8 (13.6)	85.0 (13.1)	-3.98	0.10
**SF-36 D - Social functioning**				
Control ‡	75.8 (22.5)	84.0 (18.5)	REF	
Online Only	91.2 (13.1)	92.2 (14.1)	1.14	0.80
Blended	80.0 (21.4)	81.0 (20.9)	-5.40	0.21
**SF-36 D – Physical role**				
Control ‡	59.4 (43.9)	65.6 (38.0)	REF	
Online Only	76.0 (37.9)	78.6 (37.3)	3.82	0.70
Blended	63.4 (43.8)	59.6 (45.9)	-9.42	0.30
**SF-36 D – Vitality role**				
Control ‡	59.5 (17.0)	65.3 (18.5)	REF	
Online Only	72.9 (14.5)	74.8 (16.0)	2.81	0.54
Blended	59.3 (23.4)	61.0 (22.4)	-2.98	0.48
**SF-36 D – Emotional role**				
Control ‡	80.2 (29.2)	76.0 (37.2)	REF	
Online Only	90.3 (23.1)	82.6 (35.9)	2.93	0.77
Blended	77.4 (35.2)	83.3 (35.6)	7.99	0.40
**SF-36 D – Mental Health**				
Control ‡	76.5 (13.4)	77.3 (15.9)	REF	
Online Only	86.0 (12.1)	87.8 (13.1)	5.36	0.20
Blended	74.4 (21.0)	77.0 (20.5)	1.31	0.74
**SF-36 D – General Health**				
Control ‡	61.4 (21.0)	67.5 (18.8)	REF	
Online Only	70.2 (20.2)	72.1 (21.8)	0.90	0.85
Blended	62.1 (22.1)	59.2 (22.7)	-7.73	0.09
**SF-36 D – Bodily pain**				
Control ‡	79.8 (20.1)	85.8 (17.6)	REF	
Online Only	87.1 (18.7)	87.2 (15.4)	-2.36	0.60
Blended	77. 3 (20.9)	72.5 (21.4)	-12.09	< 0.01
**Stage of change** *N* (%)				
Pre-contemplation	1 (0.8%)	2 (2.4%)		
Contemplation	6 (7.1%)	6 (7.1%)		
Preparation	26 (31%)	11(13.1%)		
Action	9 (10.7%)	13 (15.5%)		
Maintenance	42 (50%)	48 (57.1%)		
Missing	0 (0%)	6 (7.1%)		
Control ‡	3.8 (1.2)	4.0 (1.1)	REF	
Online Only	4.0 (1.2)	4.3 (1.3)	0.23	0.45
Blended	4.0 (1.1)	4.5 (0.8)	0.43	0.14
**Self-efficacy** (range 1-10)				

Control ‡	7.2 (1.9)	7.4 (1.9)	REF	
Online Only	8.0 (1.4)	8.0 (1.8)	0.13	0.75
Blended	6.9 (1.9)	7.3 (1.7)	-0.01	0.97
**Barriers** (range 1-5)				
Control ‡	2.1 (0.7)	1.8 (0.6)	REF	
Online Only	1.8 (0.5)	1.6 (0.5)	-0.12	0.28
Blended	1.9 (0.5)	1.9 (0.6)	0.16	0.11
**Benefits** (range 1-5)				
Control ‡	3.8 (0.5)	3.9 (0.6)	REF	
Online Only	3.8 (0.8)	4.2 (0.5)	0.19	0.18
Blended	3.7 (0.7)	3.8 (0.7)	-0.05	0.68
**Attitude** (range 1-7)				
Control ‡	5.5 (1.1)	6.0 (0.9)	REF	
Online Only	5.9 (0.7)	6.1 (0.9)	0.19	0.50
Blended	5.6 (1.2)	5.5 (1.1)	-0.44	0.10
**Social Support** (range 1-5)				
Control ‡	4.6 (0.7)	4.7 (0.9)	REF	
Online Only	4.8 (0.8)	4.7 (0.5)	-0.05	0.82
Blended	4.4 (0.9)	4.8 (0.7)	0.03	0.89

† AMD: Adjusted Mean Difference

‡ REF: Control is reference group.

* **Completed questionnaires at T0 and T1:** Control N=32, Online Only N=24, Blended N = 28.

A IPAQ scores represent total MET (Metabolic Equivalent of Task) minutes per week.

B MFI scores range from 4 to 20, high scores indicate high fatigue.

C POMS scores range from 1 to 5, high scores indicate more mood problems, expect form vitality, which is reversed, higher score indicate more vitality.

D SF-36 scores range 0-100, high scores indicate a better experienced quality of life.
