# Supplementary material for: Effects of and Lessons Learned from an Internet-Based Physical Activity Support Program (with and without Physiotherapist Telephone Counselling) on Physical Activity Levels of Breast and Prostate Cancer Survivors: The PABLO Randomized Controlled Trial
Source: Cancers (Basel). 2021 Jul 21;13(15):3665. doi: 10.3390/cancers13153665 (PMC8345041; doi:10.3390/cancers13153665)
Supplement: Supplementary file 1 [file cancers-13-03665-s001.zip › SupplementaryMaterial_Final.pdf]

Supplementary Material

Sa. Methods Secondary Outcomes and Cost-Analyses

All questionnaires were completed electronically, using "Exploratio" (Newcom Research & Consultancy, Amsterdam, the Netherlands).

Self-reported PA, Fatigue, Mood, and HRQOL

Self-reported PA behavior was assessed with the International Physical Activity Questionnaire (IPAQ). The IPAQ assesses four domains; PA at work, during transport, at home and during leisure time. Scores were calculated according to the IPAQ manual, resulting in MET-minutes per week, as total score per domain [30].

Fatigue was assessed with the Multidimensional Fatigue Inventory Questionnaire (MFI)[31]. The MFI consists of 20-item divided into five dimensions; general fatigue, physical fatigue, mental fatigue, reduced activity, reduced motivation. Higher scores indicate higher levels of fatigue.

Mood was assessed using the Profile of Mood States (POMS) questionnaire [32]. The 32-item POMS consists of five mood scales: anger, depression, fatigue, tension and vitality. For anger, depression, fatigue and tension, higher scores indicate poorer outcome. For vitality, higher scores indicate more vitality. Items scores range from 0 to 4. The total score was calculated by the sum of the means of the four mood scales, minus the vitality score. Higher scores indicate higher levels of anger, tension, depression and fatigue and less vitality.

HRQOL was assessed by the 36-Item Short Form Health Survey (SF-36). The SF-36 covers eight dimensions of patients' quality of life [33]. These dimensions include physical functioning, vitality, role functioning limitations due to physical problems, role functioning limitations due to emotional problems, social functioning, physical pain, mental health and general health. Higher scores indicate higher levels of functioning and quality of life.

HRQOL collected for the cost-analysis was assessed by the Euroqol EQ-5D-5L questionnaire. Total scores and clinical utility values were calculated according to the EQ-5D-5L user guide [34]. Index scores range from 0 to 1 and visual analog scale (VAS) scores range from 0 to 100. Higher scores indicate better HRQOL.

Behavioral and attitudinal variables towards PA

Based on the Transtheoretical model, current exercise behavior stage was obtained by a single item which asked respondents to choose the one statement from 5 statements corresponding to each of the stages of change (as described above) that corresponded best to their current situation [35].

Study-specific questions based on the theory of planned behavior were used to assess self-efficacy, barriers to and benefits of PA, and perceived social support [36,37].

Perceived self-efficacy regarding PA was assessed with five items. Respondents rated how likely they thought they would exercise when tired, in a bad mood, when feeling pressed for time, when on holiday, or with bad

weather, on a 0-10 response scale [34]. The overall self-efficacy score was obtained by calculating the average of all the items. A higher score indicates a stronger sense of self-efficacy. The Cronbach's α for this scale in our sample was 0.85.

Items on perceived barriers for and benefits of PA were selected from two existing questionnaires [36, 38], as previously used by Van Waart et al. [37]. Potential barriers were assessed by 18 items with a 5-point Likert-type response scale (descriptor 'never a barrier' to descriptor 'really often a barrier'), and included motivation, money, time, energy, other obligations, transportation, support for exercise, counseling about exercise, limited possibilities in the environment, pleasure, family obligations, fear of injuries, discipline, health conditions, nausea, fatigue, pain, and work responsibilities. The barrier score was calculated as the average of the item scores. Higher scores indicate a higher perceived level of barriers. The Cronbach's α of the total scale was 0.87.

Perceived benefits of PA were assessed by 11 items concerning statements of improved health leading to a reduced risk of disease, feeling better about oneself, improved fitness, improved daily functioning, weight loss, meeting new people, getting one's mind off cancer and its treatment, improved overall well-being, coping with the stress of cancer and treatment, gaining control over cancer and life, and recovering from treatment. Items were scored on a 5-point Likert-scale (descriptor 'completely disagree' to descriptor 'completely agree'). The benefit score was obtained by averaging the item scores. A higher score indicates a higher sense of benefits. The Cronbach's α for this scale was 0.91

Attitude towards PA was assessed using 7-point adjective rating scales. Two dimensions were measured: 1) instrumental attitude (useful–useless, harmful–beneficial, wise–foolish, and bad–good) and 2) affective attitude (enjoyable–unenjoyable, boring– interesting, pleasant–unpleasant, and easy–hard) [36]. The overall score for attitude was calculated as average score of the combined 8-items. The Cronbach's α of this scale was 0.95. Higher scores indicated more positive attitude towards exercise [37].

Finally, perceived social support for being physically active from partner, family, friends, colleagues, general practitioner, treating physician, and other patients with cancer was assessed. These items were scored on a 5-point Likert-type response scale (descriptor 'completely disagree to descriptor 'completely agree'), with an overall Cronbach's α of 0.90. An overall perceived support score was calculated as the average of all items [37,39]. The higher the scores the more perceived social support.

Cost-analysis

Cost analysis was performed for both interventions to give an overview of the financial impact on implementation. We assumed that an online patient-portal integrated with the Electronic Medical Record (EMR) is available to host this intervention. In the Netherlands, most hospitals have an electronic patient file software-system that, in theory, should be able to host an online patient-portal as part of the standard package system. Personnel costs for platform development, implementation, and system maintenance were based on Dutch salary scales for hospitals [40]. Costs for physiotherapist consultations were based on the Dutch reference prices [41]. All costs were

discounted to 2019 prices. Total costs for both the online only and the blended care group are listed. The price per patient dependent on the population size was graphically displayed.

Sb. Table S1: Advantages and Disadvantages of the embedding into the patient portal.

IPAS embedded in patient portal	
**Advantages**	**Disadvantages**
IPAS Care as natural part of the treatment	Technical difficulties while building
Easy access to program for physician and physical therapist	Testing and monitoring limitations during the trial because of privacy issues.
Integrated in existing patient pathway and actual link to electronic medical record	Upscaling to other hospitals complicated<br>- Costly<br>- Different timelines of project and institutional IT calendar
	Limitations in design and abilities of the program
	Two factor authentication login to patient portal can be a barrier

Table S1. Advantages and Disadvantages of the embedding into the patient portal.

Sc. Cost analysis of the PABLO interventions

Table S2. Cost analysis

		**Internet-based Physical Activity Support**		**Internet-based Physical Activity Support + Physical therapy**		
	**Description/ unit price**	**Units**	**Total**	**Units**	**Total**	**Source**
**Personnel**						
Platform development						
System development	IT-personnel, FWG55	0.3fte,3m	€3,525.30	0.3fte,3m	€3,525.30	Dutch salary scales 2020[37]
**Content check/adjustment**	Physical scientist, FWG55	0.15fte,2m	€1,290.00	0.15fte,2m	€1,290.00	Dutch salary scales 2020[37]
**System maintenance**						
System maintenance	IT-personnel, FWG50	0.1fte,12m	€4,188.00	0.1fte,12m	€4,188.00	Dutch salary scales 2020[37]

Content updates	Physical scientist, FWG55	0.1fte,12m	€5,160.00	0.1ft, 12m	€5,160.00	Dutch salary scales 2020[37]
**Subtotal personnel costs**			**€14,163.30**		**€14,163.30**	
**Total costs software costs for purchase and maintenance, per patient - if offered to 1000 patients:**			**€14.16**		**€14.16**	
**Physical therapy costs**						
Physical therapist consultation	€33.00	NA	NA	1	€35.24	Dutch reference price[38]
Physical therapist phone call	€17.00	NA	NA	5	€90.78	Dutch reference price[38]
**Total costs per patient (including software, personnel and physical therapy) - if offered to 1000 patients:**			**€14.16**		**€140.19**	

All costs are discounted to 2019 prices, including taxes. Based on the assumption that an online patient portal exists.

Table S2. Costs PABLO interventions

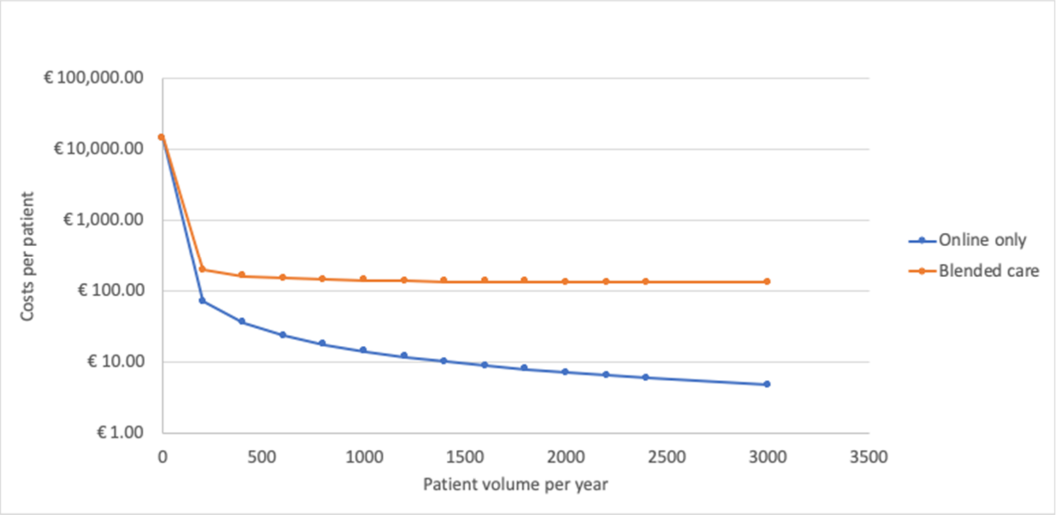

Figure S1. Total costs for both interventions calculated per patient, dependent on patient volume.

Table S3. Summary scores per tumor type.

3A. EQ-5D index scores (utilities) and EQ-5D VAS scores in **prostate cancer** in means with SD.

	N	T0				T1			
		mean index score	sd	mean VAS	sd	mean index score	sd	mean VAS score	sd
**Control**	19	0.87	0.09	71.05	24.98	0.91	0.09	79.56	11.07
**Online Only**	14	0.87	0.14	80.00	13.40	0.92	0.09	83.29	13.65
**Blended**	16	0.89	0.10	79.44	16.97	0.88	0.08	76.69	13.44
**Total**	49	0.88	0.11	76.35	19.77	0.90	0.09	79.89	12.58

3B. EQ-5D index scores (utilities) and EQ-5D VAS scores in **breast cancer** in means with SD.

	N	T0				T1			
		mean index score	sd	mean VAS	sd	mean index score	sd	mean VAS score	sd
**Control**	13	0.78	0.11	67.85	18.37	0.84	0.08	79.00	15.29
**Online Only**	10	0.85	0.18	81.10	14.88	0.88	0.11	77.14	19.28
**Blended**	12	0.80	0.10	74.08	16.98	0.80	0.20	74.83	14.23
**Total**	35	0.80	0.13	73.77	17.33	0.83	0.14	77.03	15.42
